# NLR Immune Receptors Can Exhibit Tissue-specific Expression Patterns Across Legume Species

**DOI:** 10.1093/gbe/evag257

**Published:** 2026-10-30

**Authors:** Rita Maravilha Marques, Carmen Santos, Hsuan Pai, Maria Carlota Vaz Patto, Sophien Kamoun, Jiorgos Kourelis

**Affiliations:** Instituto de Tecnologia Química e Biológica António Xavier, Universidade Nova de Lisboa, Oeiras 2780-157, Portugal; The Sainsbury Laboratory, University of East Anglia, Norwich, NR4 7UH, UK; Instituto de Tecnologia Química e Biológica António Xavier, Universidade Nova de Lisboa, Oeiras 2780-157, Portugal; National Institute for Agrarian and Veterinary Research (INIAV), Oeiras 2780-157, Portugal; The Sainsbury Laboratory, University of East Anglia, Norwich, NR4 7UH, UK; Instituto de Tecnologia Química e Biológica António Xavier, Universidade Nova de Lisboa, Oeiras 2780-157, Portugal; The Sainsbury Laboratory, University of East Anglia, Norwich, NR4 7UH, UK; The Sainsbury Laboratory, University of East Anglia, Norwich, NR4 7UH, UK; Department of Life Sciences, Imperial College London, London SW7 2AZ, UK

**Keywords:** RNA-seq, Fabaceae, phylogenomics, plant immunity, tissue expression

## Abstract

Pathogen pressure threatens legume crop productivity worldwide. Nucleotide-binding leucine-rich repeat (NLR) immune receptors serve as crucial plant resistance genes, recognizing pathogens and triggering immunity. However, the extent and patterns of NLR expression in different tissues and organs, notably across evolutionary time, remain largely uncharacterized. To investigate tissue specificity of NLR expression in the Fabaceae (legumes), we conducted comparative analyses integrating phylogenomics and transcriptomics in root and leaf tissues across different legume species. The NLR repertoires of 28 legumes were grouped into 5 monophyletic clades: coiled-coil NLR (CC-NLR), Toll/interleukin-1 receptor NLR (TIR-NLR), G10-subclade CC NLR (CC_G10_-NLR), RESISTANCE TO POWDERY MILDEW 8-like CC NLR (CC_R_-NLR), and TIR-NB-ARC-like β-propeller WD40/tetratricopeptide repeats (TNPs). Most legume NLRs belonged to CC-NLR and TIR-NLR clades, followed by CC_G10_-NLR, CC_R_-NLR, and TNP clades. In 7 of these species, comparative analysis of NLR expression in leaves versus roots revealed that over half (∼57%) of expressed NLR genes showed predominant expression in one tissue: 34% in roots (451/1336), and 23% in leaves (311/1336). We identified 324 root-specific NLRs, 171 leaf-specific NLRs, and 841 nonspecific NLRs, with an average tissue specificity per species of 32%. The closely related species grass pea (*Lathyrus sativus*) and pea (*Pisum sativum*) were an exception, showing higher levels of leaf-specific rather than root-specific NLR expression. We also identified conserved tissue expression patterns across legume species, resulting in a comprehensive resource describing tissue expression bias, enrichment, and specificity for 113 phylogenetic NLR subclasses. These legume NLR repertoires will support comparative studies between species and inform precision-breeding programs considering tissue expression patterns.

## Graphical Abstract

**Graphical Abstract:**
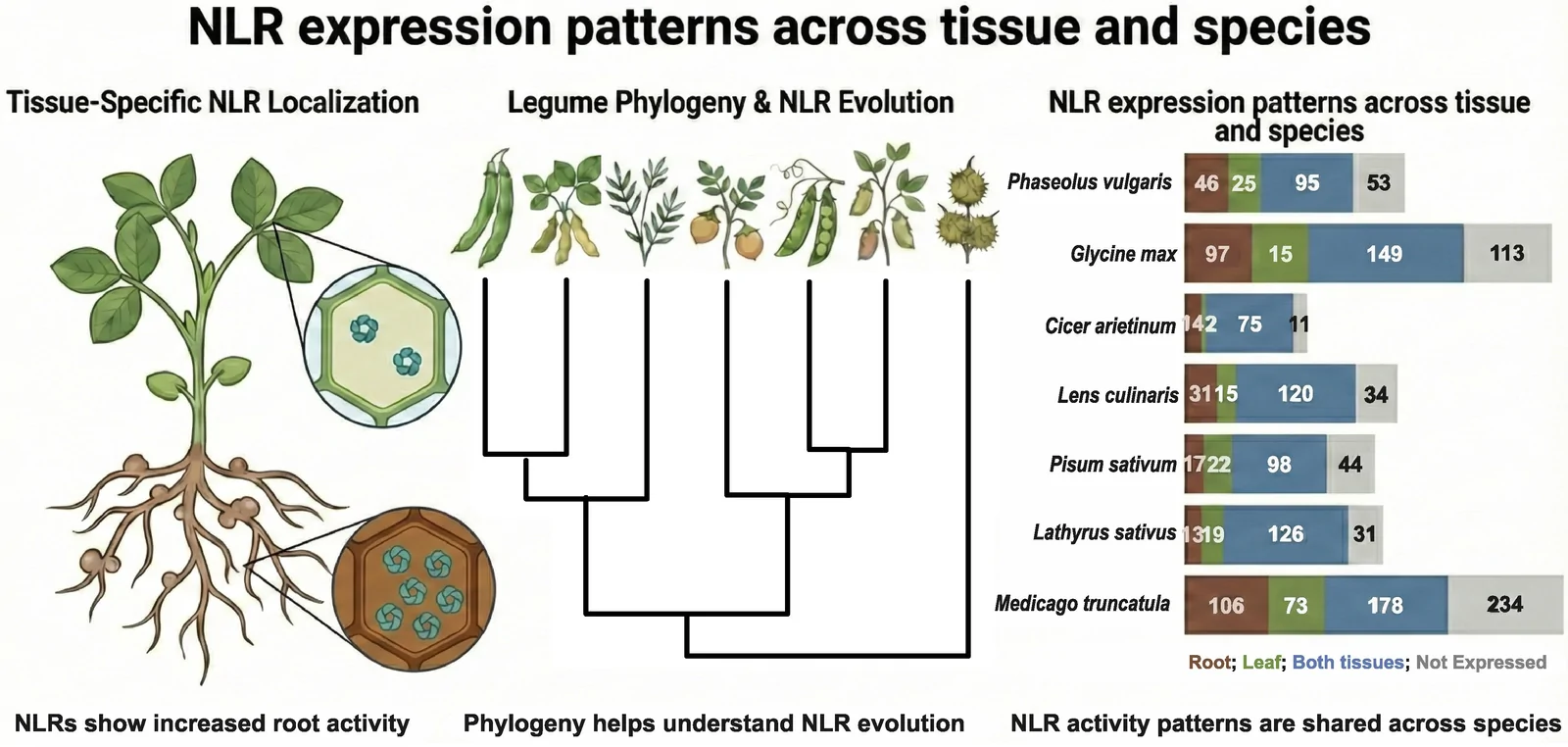

Significance Plants defend themselves from disease using immune genes called Nucleotide-binding leucine-rich repeat immune receptors (NLRs). These genes help plants detect harmful microbes and activate defenses to stop infections. In this study, we compared NLR genes across many legumes, which include crops, such as peas, beans, and lentils. We specifically examined whether these genes are more active in roots or leaves, 2 tissues that face different types of pathogens.

We found that many NLRs show strong tissue-specific activity, meaning they are mainly active in either roots or leaves. We also discovered NLR activity patterns that are shared across different species, suggesting common defense strategies. By organizing these patterns into a comprehensive resource, our work provides a clearer picture of how plant immune systems are deployed across tissues. This knowledge can help guide future research and the development of crops with improved resistance to diseases through more targeted and efficient breeding strategies.

## Introduction

The legume family comprises 765 genera and over 19,600 species, representing one of the largest and most diverse plant families (Cannon et al. 2015; Wojciechowski 2019). Annual production reaches 445 million tons of legume feed and fodder globally (Food and Agriculture Organization of the United Nations, 2022), with human consumption of pulses amounting to 101 million tons in 2024 (OECD/FAO, 2025). Legumes contain high protein levels (16% to 50%) and fix atmospheric nitrogen through symbiotic relationships, making them agriculturally and economically important while enriching soil fertility (Cannon et al. 2015). However, diseases, including mildews, rusts, blight, molds, rots, and wilts constrain legume productivity and threaten global food security, with agricultural losses from diseases reaching 45% globally (Rubiales et al. 2015; Dutta et al. 2022; Zhou et al. 2025). Chemical control remains a widely used method for disease management. However, its economic, environmental, and public health disadvantages highlight the urgent need for more sustainable pest management strategies (Zhou et al. 2025). Resistant plant varieties represent the most efficient, cost-effective, and environmentally sustainable approach to disease management (Rubiales et al. 2015). Understanding plant immunity and pathogen defense mechanisms is crucial for improving resistance breeding efficacy (Bentham et al. 2020).

Nucleotide-binding leucine-rich repeat proteins (NLRs) are among the most extensively studied and economically relevant gene families due to their importance in disease resistance (van Wersch et al. 2020). NLRs act as immune receptors, functioning in immunity by directly and indirectly detecting pathogen proteins (Förderer and Kourelis 2023). In the absence of recognition, these pathogen proteins, known as effectors, manipulate the host, allowing the pathogen to colonize and cause disease (Khan et al. 2018).

Canonical plant NLRs consist of a variable N-terminal domain, a central NB-ARC module (for nucleotide-binding APAF-1, certain plant *R* gene products, and CED-4), and a C-terminal leucine-rich repeat (LRR) domain (Van Ooijen et al. 2008). The NB-ARC module consists of 3 domains: a nucleotide-binding domain (NBD), a Helical Domain 1 (HD1), and a Winged-Helix Domain (WHD). This module contains a functional ATPase and regulates NLR activity through its nucleotide-binding state (Van Ooijen et al. 2008). As the most conserved part of an NLR, this NB-ARC module is useful for determining evolutionary relationships between NLRs through global alignments (van der Biezen and Jones 1998; Sarris et al. 2016; Kourelis et al. 2021). The LRR domain is highly variable and functions in both direct and indirect pathogen effector detection, stabilizing the active and inactive states of the NLR (Förderer et al. 2022). Beyond the canonical domains, some plant NLRs have additional “integrated domains,” which play major roles in effector recognition (Cesari et al. 2014; Białas et al. 2021).

Direct and indirect pathogen effector detection by the LRR results in a conformational switch in the NB-ARC module, allowing exchange of ADP and ATP. This results in homo-oligomerization of the NLR into either tetrameric, pentameric, or hexameric complexes known as “resistosomes” (Förderer et al. 2022). Resistosome formation activates the N-terminal domain, initiating immune signaling. Alternatively, some NLRs form pairs and networks, where one NLR is required for pathogen effector detection (sensor-NLR), while another is required for immune signaling (helper-NLR) (Adachi et al. 2019). Overall, the emerging view is to move away from a one-size-fits-all model of NLR structure and function and to appreciate the remarkable diversity of these proteins (Kourelis et al. 2021; Prigozhin and Krasileva 2021; Contreras et al. 2023a).

Based on phylogenetic trees of the NB-ARC module, flowering plant NLRs have been divided into 4 major monophyletic classes. These classes are named after their most common N-terminal domains, though individual members may lack these domains or carry non-canonical integrated domains (Barragan et al. 2021; Białas et al. 2021). N-terminal domains can include the Toll/interleukin-1 receptor/resistance (TIR) domain and 3 sequence-divergent but structurally conserved 4-helical bundle (4HB) domains (the Rx-type coiled-coil [CC] domain, the RESISTANCE TO POWDERY MILDEW 8 [RPW8]-type CC [CC_R_], and the “autonomous NLR clade”-type CC [CC_G10_]) (Förderer and Kourelis 2023). Upon activation, these canonical N-terminal domains assemble into higher-order complexes that mediate downstream immune signaling (Wang et al. 2019; Förderer et al. 2022). CC-domain-containing NLRs (CC-NLRs) are known to form pentameric or hexameric resistosomes, where the first α helix of the CC-domain forms a funnel that acts as a calcium (Ca^2+^)-permeable nonselective cation pore at the plasma membrane (Wang et al. 2019; Bi et al. 2021). Similarly, CC_G10_- and CC_R_-NLRs oligomerize upon activation and act as Ca^2+^ permeable pores (Jacob et al. 2021; Lee et al. 2021; Förderer and Kourelis 2023). Activated CC_R_-NLRs localize not only to the plasma membrane but also to organellar membranes, including the endoplasmic reticulum, chloroplast, and mitochondria (Ibrahim et al. 2026). TIR-domain-containing NLRs (TIR-NLRs) form tetrameric resistosomes upon activation, where the TIR domain displays NAD^+^ hydrolase activity, producing nucleotide-based signals that bind preformed heterodimers of the lipase-like protein ENHANCED DISEASE SUSCEPTIBILITY 1 (EDS1) with either SENESCENCE ASSOCIATED GENE 101 (SAG101) or PHYTOALEXIN DEFICIENT 4 (PAD4) (Pruitt et al. 2021; Huang et al. 2022; Jia et al. 2022). This binding induces a conformational change in the EP domain of the EDS1-SAG101 and EDS1-PAD4 heterodimers, allowing them to recruit and activate members of the CC_R_-NLR helper subfamilies N-REQUIREMENT GENE 1 (NRG1) and ACTIVATED DISEASE RESISTANCE 1 (ADR1), respectively (Dong et al. 2016; Castel et al. 2019; Wu et al. 2019, 2024; Sun et al. 2021; Huang et al. 2022; Jia et al. 2022; Yu et al. 2024).

In addition to the 4 NLR classes found in flowering plants, a fifth small, highly conserved monophyletic class called TIR-NB-ARC-like-β-propeller WD40/TPRs (TNPs) exists, which phylogenetically clusters with non-plant NLRs (Kourelis et al. 2021). These TNPs contain a phylogenetically distinct TIR domain acquired independently from TIR-NLRs (Johanndrees et al. 2023). TNPs lack the LRR of canonical plant NLRs and instead contain WD40 amino acid repeats and tetratricopeptide-like repeats (TPRs), which could serve as a ligand-binding domain (Johanndrees et al. 2023).

Because of their effector detection role, the NLR family tends to be among the most polymorphic in plant genomes, generating substantial variations in NLR sequence diversity and copy number (Zheng et al. 2016; Kourelis et al. 2021). Current NLR knowledge remains largely confined to a few model and crop species, such as *Arabidopsis thaliana*, Solanaceae, and cereals, despite the agricultural significance of Fabaceae crops (Kourelis et al. 2021; Förderer and Kourelis 2023; Contreras et al. 2023b). The complete set of NLRs within a genome (the NLRome) of certain legume species has been recently described, including soybean (*Glycine max*), common bean (*Phaseolus vulgaris*), barrel medic (*Medicago truncatula*), peanut (*Arachis hypogaea*), red clover (*Trifolium pratense*), *Lotus japonicus*, pea (*Pisum sativum*), grass pea (*Lathyrus sativus*), lentil (*Lens culinaris*), faba bean (*Vicia faba*), common vetch (*Vicia sativa*), adzuki bean (*Vigna angularis*), mung bean (*Vigna radiata*), cowpea (*Vigna unguiculata*), and narrowleaf lupin (*Lupinus angustifolius*), among other species (Qureshi et al. 2023; Rani et al. 2023; Negi et al. 2024; Sultan et al. 2024). Recovering complete NLR repertoires is difficult because NLRs are clustered, repetitive, and rapidly evolving, and these loci were often unresolved in fragmented assemblies. Resistance-gene enrichment sequencing (RenSeq), particularly when combined with long-read sequencing, was developed to capture them (Jupe et al. 2013), and telomere-to-telomere assemblies now resolve these regions directly (Mo et al. 2024). Annotating NLRs is equally difficult, because evidence-based gene prediction tends to merge tandemly arrayed paralogues, especially from short-read data, so predicting these genes ab initio without transcript evidence can be more accurate. Deep-learning predictors now match state-of-the-art evidence-based pipelines even on genes where evidence helps (Holst et al. 2026; Zhang et al. 2026) and have been applied to NLR annotation (Toghani, Sugihara and Kamoun 2026). Variation in legume NLR abundance is remarkable, even within the same genus, with NLRome size varying between 77 and 348 NLRs among *Glycine* species (Sultan et al. 2024).

Variation in NLR expression across tissues and organs is a surprisingly understudied aspect of the plant immune system (Lüdke et al. 2023). Precise regulation is critical, as the misexpression of NLRs can lead to autoimmunity and fitness costs, and it is plausible that their expression can be confined to where it is most needed (Palma et al. 2010; Richard et al. 2018; Fick et al. 2022; Lüdke et al. 2025). Roots and leaves are physiologically distinct and have fundamentally different microenvironments (Bodenhausen et al. 2013; Munch et al. 2017). Additionally, roots and leaves are exposed to different types and levels of pathogens, pests, and beneficial microbes. Interestingly, *Arabidopsis thaliana* shows predominant leaf NLR expression, while the legume *L. japonicus* shows an opposite trend toward root expression, highlighting potential differences between these species in pathogen exposure patterns (Munch et al. 2018). In other species, some NLRs display tissue specificity. For example, in tomato, the helper-NLR NLR-REQUIRED FOR CELL-DEATH (NRC) 6 and the sensor-NLRs Hero and MeR1, which provide immunity toward cyst- and root-knot nematodes, respectively, are root-specifically expressed (Lüdke et al. 2025).

Here, we integrated phylogenomic classification with transcriptomic profiling to characterize NLR tissue expression patterns across diverse legume species, generating a community resource that supports future comparative studies. We first identified and characterized the NLRomes of 28 legume species using NLRtracker and classified them into 5 monophyletic classes using the RefPlantNLR dataset as reference (Kourelis et al. 2021). We then conducted comparative transcriptomic analysis of NLR expression in leaves and roots for 7 of these species. Our study revealed that legumes show predominant NLR gene expression in roots, although both grass pea (*Lathyrus sativus*) and pea (*P. sativum*) displayed higher rates of leaf-specific expression. We investigated whether tissue specificity varied according to monophyletic NLR categories, revealing enhanced tissue specificity relative to the genome-wide average. Additionally, we observed tissue expression patterns that were conserved across species. Our findings offer insight into how spatial expression contributes to NLR function and evolution, raising the hypothesis that selective pressures shape the spatial deployment of immune receptors in plant tissues.

## Results

### Legume Phylogeny and Genome Quality Assessment

Accurate phylogenetic relationships and high-quality genomic resources are essential for comparative NLR analysis. To establish evolutionary relationships among legume species and assess resource quality, we analyzed 3 chloroplast genes and evaluated genome assembly metrics. Phylogenetic analysis of 3 chloroplast genes (*atpA*, *matK*, and *rbcL*) confirmed expected evolutionary relationships among the 28 legume species and 4 outgroups (Fig. S1a). Both monocots (*Z. mays* and *O. sativa*) clustered together, followed by the dicot outgroups (*S. lycopersicum* and *A. thaliana*), then the mimosoids (*C. canadensis* and *P. cineraria*), and finally the papilionoid clade species (Fig. S1a).

Genome quality assessment revealed substantial variation in assembly contiguity and completeness across legume resources (Fig. S1b and c; Tables S1 and S2). Fifteen of 28 legume genomes were highly contiguous (contig N50 > 1 Mbp), while 16 predicted proteomes were considered complete (<5% missing BUSCOs) (Fig. S1c; Table S2). Duplicated BUSCO scores ranged widely from 1.6% in *C. arietinum* to 95.2% in *A. hypogaea*, highlighting significant variation in genome duplication rates across legume species (Fig. S1c). Only 11 of the 32 predicted proteomes met both contiguity and completeness criteria, though all predicted proteomes were retained for subsequent analyses.

These analyses provided the phylogenetic framework for comparative studies and revealed substantial variation in genomic resource quality across the legume family.

### Legume NLR Gene Counts Exhibit Extensive Diversity and Phylogenetic Structure

Predicted NLR repertoires vary dramatically across plant species and families. To characterize legume NLR diversity and classify them into functional groups, we identified NLRs across 28 legume species and assigned them to monophyletic classes using phylogenetic analysis.

Legume NLR gene counts range from 88 in *L. albus* to 970 in *A. hypogaea* (Fig. 1a). A moderate positive correlation (*R* = 0.59, *P*-value < 0.001) exists between NLR gene counts and predicted proteome size (Fig. 1a). The proportion of predicted protein-coding loci encoding NLRs varied widely, from 0.12% in *L. albus* to 2.27% in *P. alba* (Fig. 1b). Three legume species (*M. truncatula*, *A. hypogaea*, and *P. alba*) contained up to 3 times as many NLRs as expected for their predicted proteome size, harboring 645 to 931 predicted NLRs (Fig. 1b). These species contrasted sharply with *C. arietinum*, *V. angularis*, *Aeschynomene evenia*, *P. sativum*, *P. lunatus*, *C. cajan*, *L. albus*, and *L. angustifolius*, which had fewer predicted NLRs than expected (Fig. 1b).

**Fig. 1.**
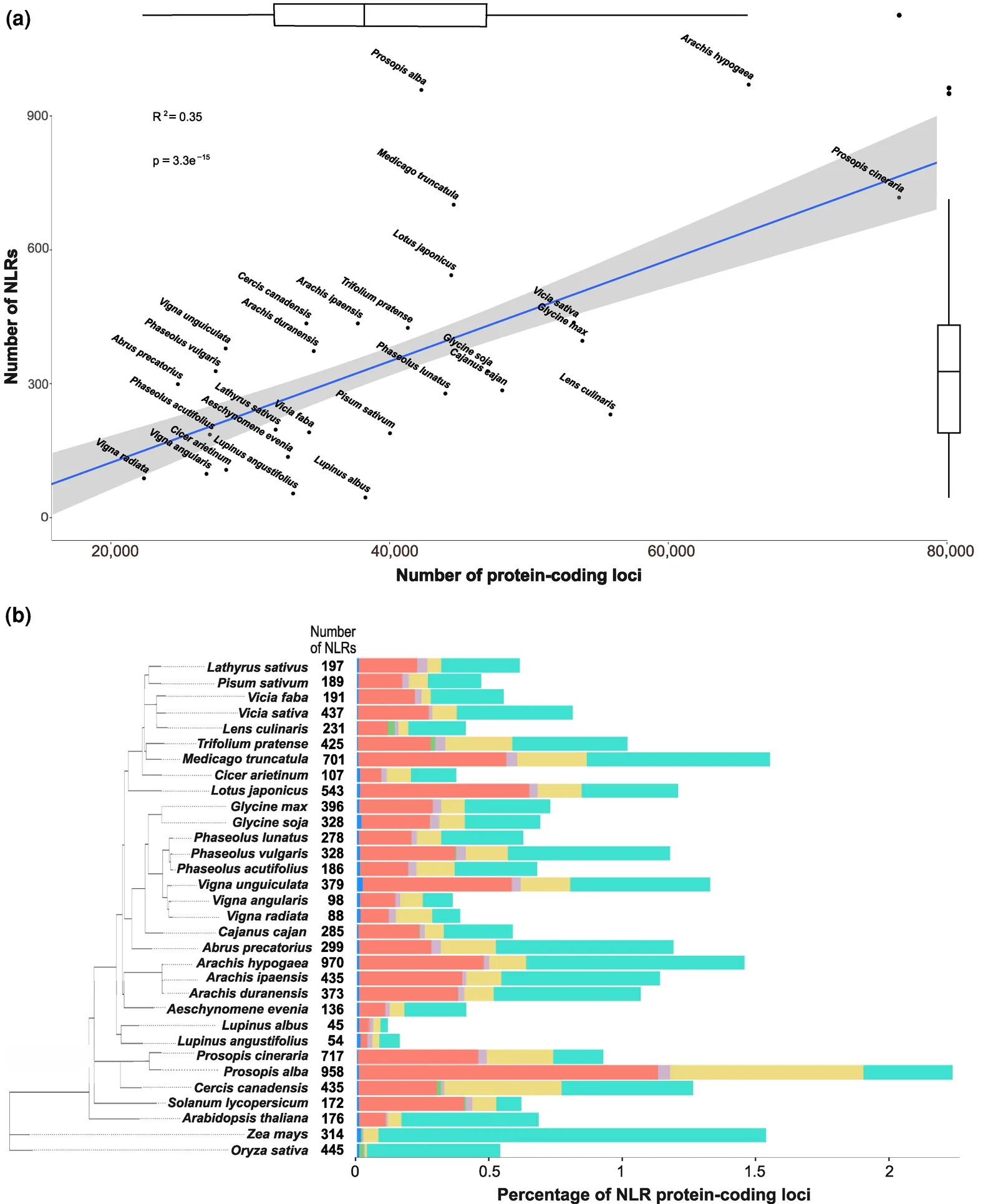
Legume NLR gene loci and counts. (a) NLR gene counts correlate moderately with predicted proteome size across legume species. Scatter plot showing NLR gene number versus protein-coding loci for each species. (b) NLR class distribution varies across legume phylogeny. Percentage of NLR-coding loci for each legume and outgroup species, arranged by phylogenetic relationships using a cladogram based on chloroplast genes (*atpA*, *matK*, and *rbcL*). Numbers indicate total NLR-coding protein loci per genome. Color coding: turquoise (CC-NLRs), yellow (CC_G10_-NLRs), lilac (CC_R_-NLRs), salmon (TIR-NLRs), blue (TNPs), and green (NLRs that did not cluster with monophyletic clades present in the RefPlantNLR dataset).

NLR classification through NB-ARC domain alignment with RefPlantNLR dataset revealed clustering into canonical flowering plant NLR monophyletic clades. CC-NLRs predominated across species, followed closely by TIR-NLRs, then CC_G10_-NLRs, with CC_R_-NLRs and TNPs being least abundant (Fig. 1b, Fig. S2). Six species (*O. sativa*, *Z. mays*, *A. thaliana*, *P. vulgaris*, *T. pratense*, and *L. culinaris*) contained NLR groups that failed to cluster with reference NLRs and were classified as “Other” (Fig. 1b). NLR class distribution largely reflected interspecific phylogenetic relationships, with legume NLR profiles showing greater similarity to dicot outgroups than to monocots (Fig. 1b). Monocot outgroups (*O. sativa* and *Z. mays*) completely lacked TIR-NLRs. *V. radiata* and the early-diverging legumes *P. cineraria*, *P. alba*, and *C. canadensis* showed overrepresentation of CC_G10_-NLRs relative to other species (Fig. 1b).

Legume NLRomes exhibit extensive diversity in gene counts and phylogenetic structure, with class distributions reflecting evolutionary relationships.

### Tissue Identity Accounts for Greater Expression Variation Than Species Differences

Understanding the relative contributions of tissue type versus species identity to gene expression patterns is crucial for interpreting tissue expression analyses. To determine the primary sources of expression variation in legumes, we performed transcriptomic analysis across 7 species in 2 tissue types. It is noteworthy that these seedlings showed root nodules upon harvest, regardless of species.

Transcriptomic analysis of BUSCO fabales orthologues across leaf and root tissues revealed tissue as the primary source of expression variation (Fig. 2a). Principal component analysis of RNA samples showed clear clustering of biological replicates (Fig. S3a). The first principal component (PC1) separated samples by tissue and accounted for 70% of total expression variation, while PC2 separated samples primarily by species phylogeny and explained only 5% of variation (Fig. S3a). Leaf and root expression patterns clustered first by tissue, then by species (Fig. S3a). Leaf samples displayed greater variability than root samples (Fig. S3a), and on PC2, species separated into 2 phylogenetic clusters: the Phaseoloids (*G. max* and *P. vulgaris*) plus *M. truncatula*, and the remaining Hologalegina species (*C. arietinum*, *L. culinaris*, *P. sativum*, and *L. sativus*) (Fig. S3a).

**Fig. 2.**
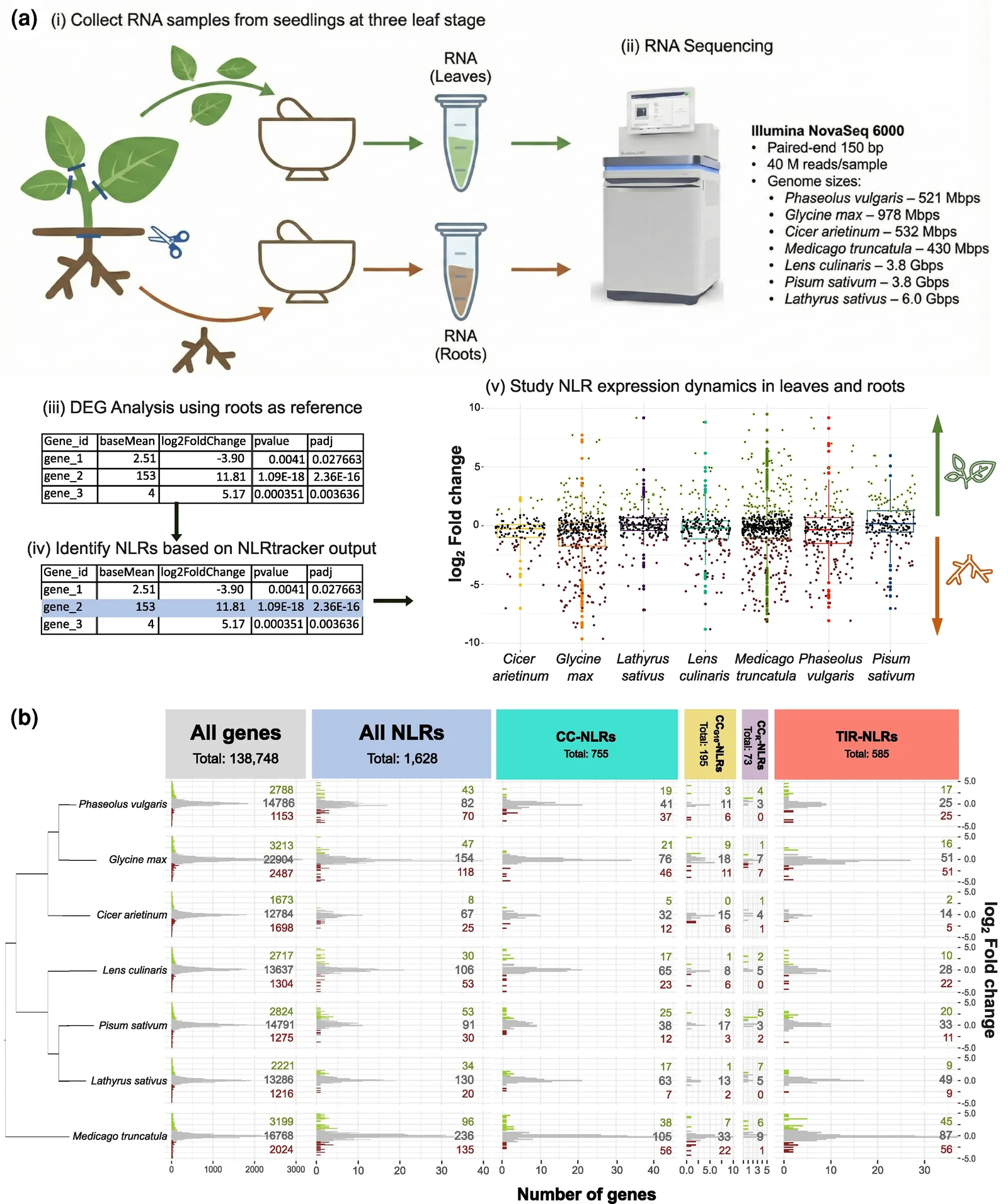
Tissue-specific expression patterns in legume transcriptomes. (a) Experimental design showing tissue collection from seedlings at the 3-leaf developmental stage for RNA-seq analysis using Illumina NovaSeq 6000 (150 bp paired-end, 40 M reads per sample), followed by DEG analysis, NLR identification, and analysis of NLR expression dynamics. (b) Tissue expression profiles across 7 legume species. Horizontal histograms show gene frequency (*x* axis) versus log_2_ fold change (*y* axis). Positive values indicate leaf-predominant expression (green), negative values indicate root-predominant expression (brown). Absolute gene numbers are shown on the right. The total shown for each category is the number of expressed (detectable) genes. Over-representation of differentially expressed genes among NLRs relative to all genes was assessed with a 2-tailed Fisher's exact test (common odds ratio 3.2 across species by Cochran–Mantel–Haenszel, *P* < 0.001).

Clustered heatmap analysis further supported this tissue-dependent pattern, with samples clustering primarily in 2 tissue groups, with closely related species forming distinct clusters within each group (Fig. S3b). Differences between species were sufficiently large for the biological replicates from each species to cluster independently (Fig. S3b). Of 3,782 BUSCO orthologues present across all 7 species, 3,160 (84%) were expressed in both tissues. Notably, 1,075 of these genes demonstrated high average expression levels, exceeding 250 TMM.

Among the genes with highest leaf enrichment, we found *P. sativum*'s chloroplastic ribulose bisphosphate carboxylase/oxygenase activase (RuBisCO activase, LOC127083843, logFC = 15.06), *M. truncatula*'s chloroplastic carbonic anhydrase (MtrunA17_Chr6g0450641, logFC = 14.78), *C. arietinum*'s ribulose-bisphosphate carboxylase (RuBP carboxylase, Ca_16979, logFC = 14.54), *L. culinaris*' chloroplastic RuBisCO activase (Lcu.2RBY.3g039110, logFC = 14.34), *L. sativus*' chloroplastic RuBisCO activase (g19754, logFC = 14.13), *P. vulgaris*' RuBP carboxylase (Phvul.004G064800, logFC = 13.33), and *G. max*'s light-harvesting complex II chlorophyll a/b binding protein 3 (LHCB3, Glyma.13G282000, logFC = 12.19).

On the other hand, the genes with the highest root enrichment were a *P. sativum* leghemoglobin (LOC127121418, logFC = −14.14), a *M. truncatula* nodulin-25 (MtrunA17_Chr3g0100391, logFC = −14.14), a *P. vulgaris* linoleate 9S-lipoxygenase-like gene (Phvul.005G156800, logFC = −12.97), the *L. sativus* gene g29944 (no predicted gene function or domains, logFC = −12.61), the highly root-expressed (according to Phytozome data; Goodstein et al. 2012) *G. max* gene Glyma.09G092700 (logFC = −12.12), a *L. culinaris* disease resistance response protein Pi49-like (Lcu.2RBY.2g016700, logFC = −10.86), and a *C. arietinum* chitinase (Ca_18462, logFC = −9.95).

The top differentially expressed genes in leaves and roots are well-known tissue-specific markers, supporting the robustness of the transcriptomic data.

Tissue identity drives substantially greater expression variation than species differences, providing a framework for interpreting tissue expression patterns of genes.

### NLRs Exhibit Enhanced Tissue Specificity With Predominant Root Expression

NLR tissue-specificity patterns may differ from genome-wide patterns due to their specialized immune function. To investigate NLR tissue specificity compared to other gene families, we analyzed differential expression patterns across the 7 legume species.

Among 138,748 expressed genes across the 7 species, 21.47% exhibited differential expression between tissues (Fig. 2b—All genes), with leaf expression favored 1.68 to 1 (Table S8). Transcriptomic NLR analysis revealed that 1,628 of 1,858 predicted NLRs were expressed in at least one tissue, with 1,336 sufficiently expressed transcripts for differentially expressed gene (DEG) analysis (71.9% expression rate). Remarkably, 57.04% of expressed NLRs showed differential expression between tissues (Fig. 2b—All NLRs), with root expression favored 1.45 to 1 (Table S8). Legume NLRs displayed 2.66-fold greater tissue-dependent expression than the genome-wide average and reversed the general leaf-biased tendency, showing predominant root expression instead. Consistent with this, NLRs were significantly over-represented among genes differentially expressed between tissues relative to the genome-wide background (per-species 2-tailed Fisher's exact tests combined across species with a Cochran–Mantel–Haenszel test, giving a common odds ratio of 3.2 (95% CI 2.9–3.6, *P* < 0.001); enrichment was significant in all 7 species, with per-species odds ratios of 1.6–5.3 and Benjamini–Hochberg-adjusted *P* ≤ 0.0043; Table S8).

Root-specific NLR expression exceeded leaf-specific expression, particularly in *M. truncatula*, *G. max*, and *P. vulgaris* (Fig. 2b, Fig. S4). However, both *L. sativus* and *P. sativum* showed a tendency toward NLR leaf-specific expression instead (Fig. 2b, Fig. S4). On average, 21% of expressed NLRs were root-specific, with *M. truncatula* showing the highest proportion (30%) and *L. sativus* the lowest (8%) (Fig. 3b). Leaf-specifically expressed NLRs averaged 11.5% across species, with *P. sativum* displaying the highest proportion (16%) and *C. arietinum* the lowest (2%) (Fig. 3b). Most expressed NLRs (50% to 83%) were detected in both tissues across all species (Fig. 3b).

**Fig. 3.**
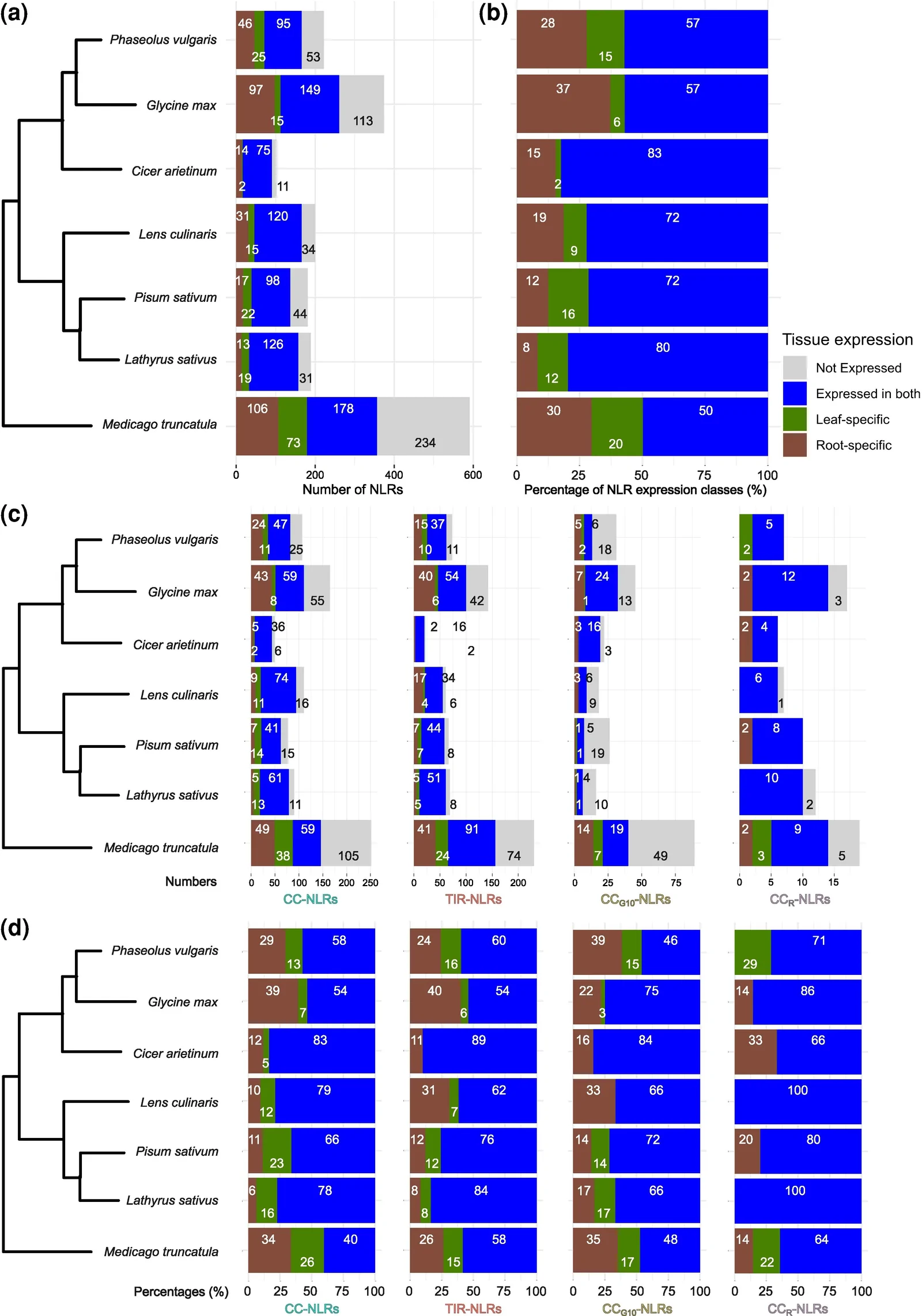
Stacked bar charts showing NLR expression patterns across tissue and species. (a) Tissue expression patterns of NLRs in absolute numbers. (b) Percentages of tissue expression patterns across expressed NLRs. (c) Stacked bar charts showing tissue expression patterns of each NLR class (CC-NLRs, TIR-NLRs, CC_G10_-NLRs, and CC_R_-NLRs). (d) Percentages of tissue expression patterns across the classes of expressed NLRs (CC-NLRs, TIR-NLRs, CC_G10_-NLRs, and CC_R_-NLRs). All figures share the same legend: Gray bars indicate non-expressed NLRs; blue bars show NLRs expressed in either tissue (non-tissue-specific); brown bars show root-specific NLRs; and green bars show leaf-specific NLRs.

NLR class-specific analysis revealed distinct tissue predominance among expressed NLRs (Table S8). CC_R_-NLRs showed leaf bias with 40% (27/67) expressing predominantly in leaves versus 19% (13/67) in roots, particularly in *L. sativus*, *P. sativum*, and *P. vulgaris* (Table S8). TNPs demonstrated strong root bias with 86.7% (13/15) showing root-specific expression (Table S7). CC_G10_-NLRs favored roots with 45.2% (57/126) root specificity versus 21.4% (27/126) leaf specificity (Table S8). CC-NLRs and TIR-NLRs showed similar patterns with ∼25% to 26% leaf specificity (158/616 and 130/512) and ∼32% to 35% root specificity (198/616 and 181/512) (Fig. 2b). Surprisingly, TIR-NLRs and their required CC_R_-NLR helper-NLRs (NRG1 and ADR1 subfamilies) showed opposing tissue predominance. While most TIR-NLRs were root-specific, their helper-NLRs were predominantly expressed in leaves.

*Medicago truncatula* and *C. arietinum* were on opposite sides of the NLRome size spectrum. *Cicer arietinum*, the species with the smallest NLRome, showed the lowest proportion of overall non-expressed NLRs (11%) and tissue-specific NLRs (17%; Fig. S4e). On the other hand, *M. truncatula*, the species with the largest NLRome, exhibited the highest proportions of non-expressed NLRs (40%) and tissue specificity (50%; Fig. S4e).

NLRs exhibit enhanced tissue specificity relative to the genome-wide average and show predominant root expression that contrasts with genome-wide leaf bias patterns.

### Evolutionary Conservation of Tissue Expression in NLR Subclasses

Tissue-enriched NLR subclasses can reveal evolutionary conservation and identify potential functional groups. To identify conserved tissue-enriched NLR subclasses, we examined prominent phylogenetic subclasses spanning NLRs from multiple species and assessed whether these groups exhibited consistent leaf- or root-enriched expression patterns (Fig. 4).

**Fig. 4.**
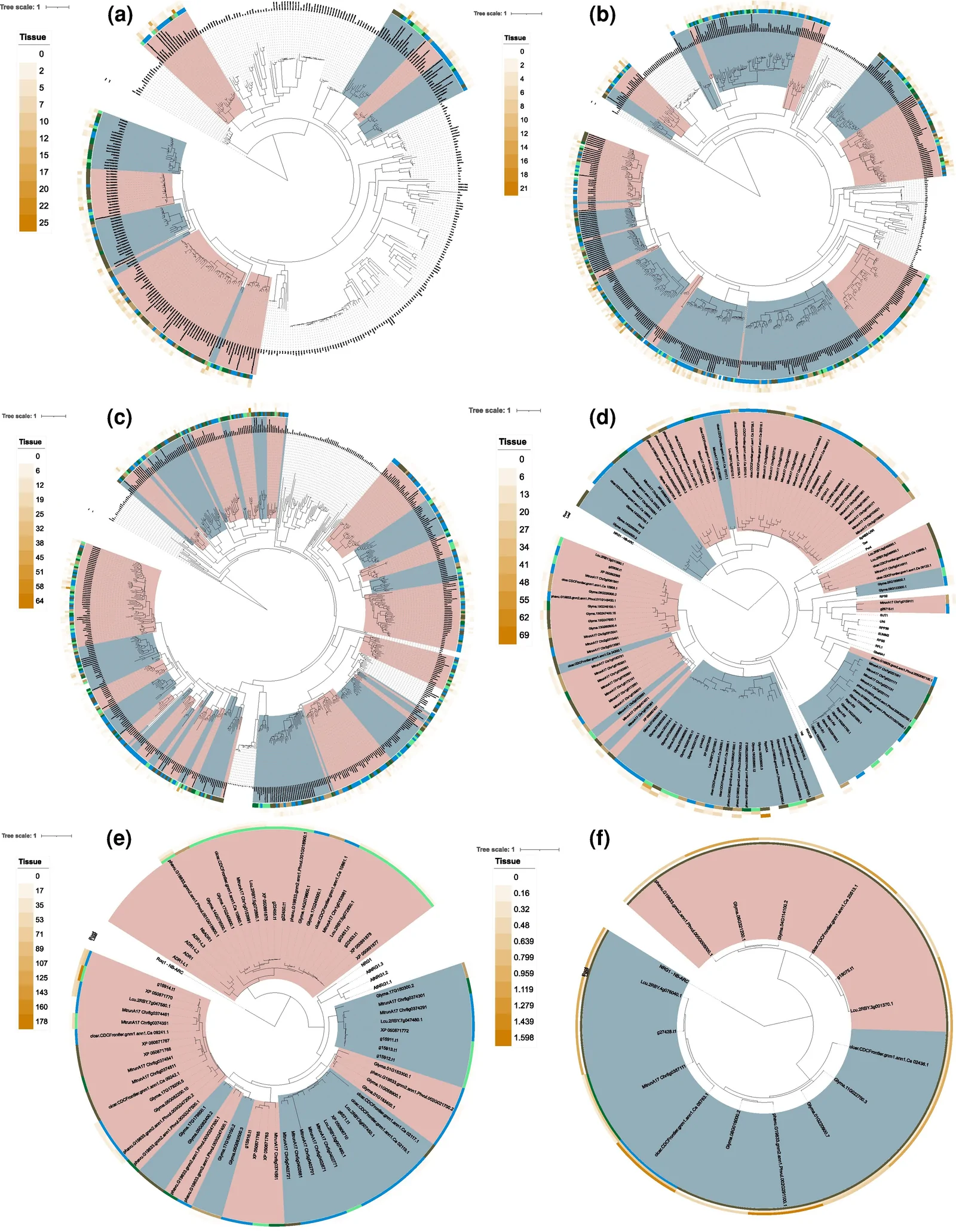
Phylogenetic trees of cross-species NLR subclasses including RefPlantNLR entries. (a) Phylogenetic tree of 164 CC-clade-1 NLRs divided into 12 subclasses. (b) Phylogenetic tree of 437 CC-clade-2 NLRs divided into 21 subclasses. (c) Phylogenetic tree of 506 TIR-NLRs divided into 51 subclasses. (d) Phylogenetic tree of 112 CC_G10_-NLRs divided into 17 subclasses. (e) Phylogenetic tree of 67 CC_R_-NLRs divided into an ADR1 subclass and 8 NRG1 subclasses. (f) Phylogenetic tree of 15 TNPs divided into 2 subclasses. The clade colors separate NLR subclasses within each NLR class phylogenetic tree. Figure generated using iTOL v6 (Letunic and Bork 2024).

Our analysis revealed 112 NLR subclasses across the 5 NLR classes, plus one subclass with NLRs classified as “Other,” totaling 113 NLR subclasses (Table S10). Among these, 12 had strong cross-species enrichment signatures, spanning CC_R_-, CC-, and TIR-NLRs. Among helper-NLRs, ADR1-01 displayed conserved leaf specialization, comprising 19 NLRs across all 7 species, of which 15 were leaf-enriched. Multiple CC-NLR subclasses also showed consistent expression bias. CC-clade1-07 was strongly root-enriched (25 root-enriched of 42 NLRs across all 7 species). CC-clade1-10 contained 16 NLRs across 4 species, with 12 showing root enrichment, whereas CC-clade1-11 included 17 NLRs across 2 species, of which 11 were root-enriched. In contrast, CC-clade1-12 represented a leaf-biased CC-NLR clade (13 leaf-enriched from 19 NLRs across 4 species). An additional CC-NLR group, CC-clade2-06, showed near-uniform root enrichment (6 of 7 NLRs across 4 species).

TIR-NLR subclasses likewise exhibited conserved tissue patterns (Table S10). Several TIR-NLR subclasses showed predominant root enrichment: TIR-02 (6 root-enriched of 10 NLRs across 5 species), TIR-11 (14 root-enriched of 22 NLRs across all 7 species), TIR-38 (3 of 5 NLRs across 5 species), and TIR-43, a large root-biased lineage composed of 32 NLRs across 2 species, with 21 showing predominant root enrichment. Leaf-biased TIR-NLR subclasses were also present, including TIR-05 (with 3 of 4 leaf-enriched NLRs across 4 species) and TIR-19 (8 leaf-enriched of 9 NLRs across 6 species).

Overall, the recurrence of tissue-enriched expression within phylogenetically close NLR subclasses indicates that leaf- or root-associated specialization is not randomly distributed but can reflect evolutionary patterns shared across legume species. These conserved subclasses provide a focused set of candidate lineages for downstream functional or evolutionary analyses.

### Tissue-specific NLRs Cluster by Species Phylogeny and NLR Class

Tissue-specific gene expression patterns may be evolutionarily conserved or may evolve independently in different lineages. To further investigate whether tissue-specific NLR expression patterns are phylogenetically conserved, we performed phylogenetic analysis of tissue-specific NLRs across 7 species.

Phylogenetic analysis of leaf-specific (Fig. 5a) and root-specific (Fig. 5b) NLRs revealed clustering patterns driven by NLR class and interspecific phylogenetic distance. NLRs from Hologalegina species (*M. truncatula*, *C. arietinum*, *L. culinaris*, *P. sativum*, and *L. sativus*) cluster together, while Phaseoloids (*P. vulgaris* and *G. max*) form separate clusters (Fig. 5). Root-specific NLRs showed markedly higher expression levels than leaf-specific NLRs (Fig. 5). Many clusters lacked representatives from some species, indicating either species-specific expansions, gene loss, or incomplete genomic resources. Root-specific CC_R_-NLRs belonged to the NRG1 subfamily, with homologs identified in 4 species (Table S9). TNPs showed root-specific expression in 5 species, whereas *M. truncatula* was the only species with a leaf-specific TNP (Fig. 5a, Table S9).

**Fig. 5.**
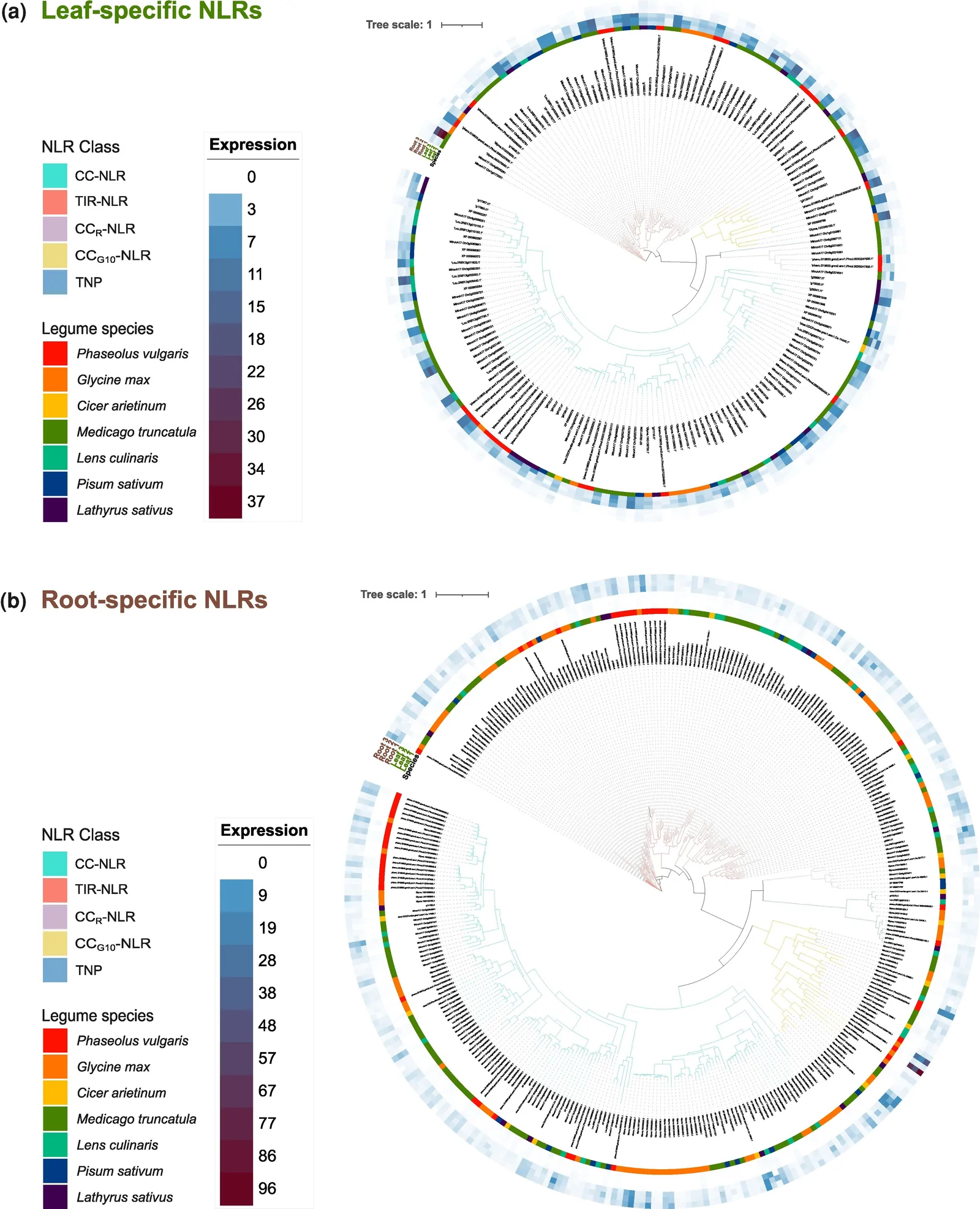
Phylogenetic relationships and expression patterns of tissue-specific NLRs. Expression values in trimmed mean of M-values (TMM) across 3 biological replicates for (a) leaf-specific and (b) root-specific NLRs. Species colors: *P. vulgaris* (red), *G. max* (orange), *C. arietinum* (yellow), *M. truncatula* (green), *L. culinaris* (teal), *P. sativum* (blue), *L. sativus* (purple). NLR class colors are represented in the color of tree branches: turquoise (CC-NLRs), salmon (TIR-NLRs), lilac (CC_R_-NLRs), yellow (CC_G10_-NLRs), and blue (TNPs). Expression scale: white (0 TMM), blue (10 TMM), dark red (respective maximum values). Figure generated using iTOL v6 (Letunic and Bork 2024).

## Discussion

In this study, we demonstrate that the expression of NLR immune receptors in legumes can be highly compartmentalized by organ tissue and provide a valuable community resource to support interspecific comparative studies. Legume NLRs exhibit over twice the level of tissue-specific expression compared to the genomic average, generally with a skew toward root tissues. This finding contrasts with the overall leaf-biased expression of most other genes, suggesting that distinct selective pressures in the rhizosphere may have shaped the evolution of the legume NLR immune system. This root-centric defensive investment in legumes aligns with observations in *Lotus japonicus* and monocots (Tarr and Alexander 2009; Munch et al. 2018), but is opposite to the shoot-skewed NLR expression reported in Brassicaceae, a group that lost the capacity for root endosymbiosis with mycorrhizal fungi (Lai and Eulgem 2018; Munch et al. 2018), highlighting potential divergence in immune compartmentalization between these lineages. The tissue deployment of distinct NLR classes, such as the predominant expression of CC_R_-NLRs in leaves and TNPs in roots, further points to the spatial organization of plant defense.

The root bias in NLR expression may reflect an evolutionary adaptation to the complex and microbe-rich soil environment (Munch et al. 2018). Roots face constant exposure to a diverse array of soil-borne pathogens and microorganisms, requiring a large and varied immune receptor repertoire. Furthermore, the unique symbiotic relationship between legumes and nitrogen-fixing rhizobia imposes an additional layer of complexity (Zhao et al. 2021). The immune system allows plants to differentiate between pathogens and beneficial microbes, and NLRs are known to act as regulators of host–rhizobia specificity (Yang et al. 2010; Kisseleva 2014; Quilbé et al. 2021). The abundance of root-expressed NLRs may therefore provide a molecular toolkit not only for pathogen defense but also for modulating symbiosis. The identification of root-specific NLRs clustering with receptors known to confer resistance to soil-borne diseases, such as Rps1-k (*Phytophthora sojae* root rot), supports their role in subterranean defense and provides promising candidates for future functional validation (Gao et al. 2005). It is also noteworthy that the present NLR expression patterns were observed in plants grown in non-aseptic conditions, with roots containing nodules, and were therefore shaped by responses to microbes present in the phyllo- and rhizosphere.

While root-biased expression is the dominant trend, our findings reveal important exceptions that underscore the influence of lineage-specific evolution. The closely related species *P. sativum* and *L. sativus* displayed predominantly leaf-specific NLR expression, opposite to the other 5 species analyzed. Since nodulation was similar across all species at the time of tissue collection, this distinct pattern is unlikely to reflect differences in symbiotic status and instead suggests that tissue-specific expression patterns can be conserved within lineages. The distinct pattern of these Fabeae tribe species (Schaefer et al. 2012; Santos and Leitão 2024) may reflect adaptation to historical or ongoing pressure from phyllosphere-specific pathogens (Rubiales et al. 2015). It could also have resulted from a depletion of NLRs expressed in roots to allow symbiosis with a wider diversity of rhizobia (Benezech et al. 2020).

Additionally, we observed a striking discordance in tissue predominance between TIR-NLRs, which were largely root-specific, and their canonical CC_R_-NLR helper-NLRs (*NRG1/ADR1* families), which were predominantly expressed in leaves and tend to be constitutively expressed. This pattern suggests 2 possible scenarios: distinct pools of helper-NLRs may operate in different tissues, with a subset sufficient to support TIR-NLR-mediated immunity in each location; alternatively, a subset of broadly expressed helper-NLRs may be sufficient to support TIR-NLR function across the entire plant, representing an efficient allocation of defensive resources (Jubic et al. 2019). The high expression values observed for CC_R_-NLRs relative to other NLR classes are consistent with their role as central, conserved hubs in immunity that must be readily available for signal transduction (Castel et al. 2019; Jubic et al. 2019; Sun et al. 2021; Contreras et al. 2023b).

Our analysis of NLRome size in relation to expression patterns reveals a potential evolutionary trade-off. Species with the largest NLRomes, such as *M. truncatula*, also exhibited the highest proportion of both non-expressed and tissue-specific NLRs. Conversely, *C. arietinum*, with one of the smallest NLRomes, showed broad expression across tissues with minimal tissue specificity. This suggests that NLRome expansion may impose a fitness cost (Richard et al. 2018) that results in an increased compartmentalization. Consequently, larger NLRomes appear to display transcriptional silencing or strict tissue-specific regulation, whereas smaller, more streamlined NLRomes may rely on widespread expression to provide protection (Richard et al. 2018). This aligns with the “birth-and-death” model of NLR evolution, where expansions are coupled with functional specialization and regulatory refinement (Michelmore and Meyers 1998; Wu et al. 2017). This pattern may also be influenced by agricultural history; the smaller NLRome in a domesticated crop that suffered major genetic bottlenecks, like *C. arietinum* (Abbo et al. 2003; Raina et al. 2019), could be a consequence of “NLRome erosion,” a process where intense selection for yield and other traits during breeding leads to the inadvertent loss of disease resistance genes that are more abundant in wild relatives (Meyer et al. 2012; Flint-Garcia 2013; Zheng et al. 2016; Ma et al. 2019; Gasparini et al. 2021; Bayer et al. 2022; Asif et al. 2023).

Constitutive NLR expression across major plant organs and tissues may reflect an adaptive strategy for this fast-evolving gene superfamily (Zheng et al. 2016; Rani et al. 2023), as expression in multiple tissues could maximize the potential benefits of any gain-of-function mutations. Tissue-restricted expression also carries an evolutionary cost. A new recognition specificity confers a fitness benefit, and can therefore be selected, only if the receptor is expressed at the site of infection by the pathogen it detects. Confining that receptor to a single organ could lower its autoimmune and metabolic cost, but it also limits the receptor to the pathogen pressure of that organ, narrowing the selection that can act on new specificities and so reducing the receptor's evolvability and the breadth of pathogens it can come to recognize. Because plants have no circulating immune cells, every cell must be immune-competent and each organ must carry the receptors that defend it, so maintaining the capacity to evolve new recognition across the whole plant demands a separate, independently diversifying set of receptors in each tissue. This trade-off predicts that NLRs under active diversification should be expressed where selection can act on them rather than transcriptionally minimized, and some evidence supports this: in *Arabidopsis*, highly variable NLRs are expressed more highly than their conserved paralogues (Sutherland et al. 2024), and functional resistance genes tend to be expressed at higher levels (Brabham et al. 2025). Unlike these rapidly diversifying sensors, the conserved helper NLRs NRG1 and ADR1 are broadly expressed because they act downstream of many sensor NLRs throughout the plant; because these helpers are ancient, dedicated mechanisms have evolved to restrain the autoimmune risk of their broad expression, as exemplified by the *Arabidopsis* negative regulator NRG1C (Wu et al. 2022; Huang et al. 2025; Xiao et al. 2025). Furthermore, the legume family experienced several whole-genome duplications across its evolution and domestication, accompanied by purifying selection forces (Zheng et al. 2016; Zhao et al. 2021). The genome dynamics in the legume family could explain the expansion and contraction of NLR repertoires across species (Zheng et al. 2016; Qureshi et al. 2023). Despite the variable quality of the legume genomic resources, we observed a significant positive correlation between proteome size and NLR number comparable to the one seen in Baggs et al. (2017), but contrasting with similar studies on legume NLRomes (Qureshi et al. 2023; Negi et al. 2024). The number of NLRs per species identified in the present study is substantially less (often around a third) than those reported in Negi et al. (2024), likely because of the strict filtering we employed to avoid double counting splicing variants and masking truncated NLRs that lack canonical domains. Nevertheless, the proportion of protein-coding loci encoding NLRs aligns with those obtained in Ngou et al. (2022).

This study establishes a framework for understanding the spatial deployment of the legume immune system across organs and tissues, while also serving as a community resource and springboard for future investigation, including comparative interspecific studies on NLR expression and breeding for tissue-specific disease resistance. The variable quality of some publicly available legume genomes may have limited the complete identification of all NLRs, and future work with improved assemblies will refine these NLR repertoires. Our analysis was conducted on unchallenged seedlings at a single developmental stage. Because we profiled a single accession and one set of growth conditions per species, our data do not capture within-species variation in NLR expression, which can be substantial (Prigozhin et al. 2025; Wei et al. 2025). Transcriptomic profiling across different life stages and in response to pathogen infection (like the studies in Fick et al. 2022, and Maravilha et al. 2025) is needed to capture the dynamic nature of NLR expression. Finally, the NLR subclasses and clades identified here, especially the ones showing tissue expression conservation across species, are prime targets for functional characterization. Validating their roles in disease resistance will be crucial for translating these findings into applied breeding programs, enabling the development of durable, bioengineered resistance targeted to the tissues where pathogenic challenges are anticipated.

## Materials and Methods

### Species Selection and Genome Acquisition

We analyzed the predicted proteomes of 28 legume species representing 6 major clades: Hologalegina, Phaseoloid, Dalbergioids, Genistoids, Mimosoids, and Caesalpinioideae (Table S1). Four outgroup species with well-characterized NLRomes were included for comparative analysis: *Arabidopsis thaliana* (Ecotype Col-0; GCF_000001735.4; Annotation by TAIR and Araport), *Solanum lycopersicum* (cv. Heinz 1706; GCF_000188115.5; NCBI Solanum lycopersicum Annotation Release 103), *Oryza sativa* (Japonica Group, strain Nipponbare; GCF_001433935.1; NCBI Oryza sativa Japonica Group Annotation Release 102), and *Zea mays* (cv. B73; GCF_902167145.1; NCBI Zea mays Annotation Release GCF_902167145.1-RS_2025_02).

Genome assemblies and proteomes were obtained from multiple repositories. From the GigaScience Database (GigaDB): *Vicia sativa* (wild accession from South Australia, GCA_021764765.1) and *Cercis canadensis* (wild accession from Iowa, United States of America, GCA_003255065.1). From the Legume Information System (LIS): *Lotus japonicus* (ecotype Gifu, GCA_012489685.2), *Lupinus albus* (cultivar Amiga, GCA_009771035.1), *Lupinus angustifolius* (genotype Tanjil, GCA_001865875.1), *Phaseolus acutifolius* (wild accession W6 15578, *Phaseolus acutifolius* WLD v2.0), *Phaseolus lunatus* (accession G27455, bioproject PRJNA596114), *Vigna angularis* (genotype Gyeongwon IT213134, GCA_000465365.1), and *Vigna radiata* (genotype VC1973A, GCA_000741045.2). *Prosopis cineraria* (wild accession growing in 24°17′18.3″ N 55°43′36.2″ E) was obtained from Zenodo (https://zenodo.org/record/6720540). *Lens culinaris* genome assembly (CDC Redberry; Lcu.2RBY) was obtained from the authors of Haile et al. (2021). The remaining genomes were retrieved from the National Center for Biotechnology Information (NCBI) platform. Complete source information and genome quality metrics are provided in Table S1.

### Phylogenetic Analysis of Legume Species

Phylogenetic relationships among analyzed species were determined using 3 chloroplast genes: ATP synthase subunit alpha (*atpA*), maturase K (*matK*), and the large subunit of ribulose bisphosphate carboxylase (*rbcL*) (Ohashi, Tateishi and Donovan Bailey 2001; Chebet et al. 2022). Gene sequences were aligned using Clustal Omega v1.2.2 in Geneious Prime v2022.2.2 (https://www.geneious.com) with default parameters (group sequences by similarity, use fast clustering with mBed algorithm, and mBed guide trees cluster size of 100). A consensus phylogenetic tree for 28 legume species was constructed using the Geneious Tree Builder with the Tamura-Nei neighbor-joining method, *Z. mays* as outgroup, and 10,000 bootstrap resamples.

### Genome Quality Assessment

Genome quality was evaluated using 2 metrics: contiguity and completeness. Contiguity was assessed using contig N50 size, with genomes having N50 ≥ 1 Mbp considered high quality (Astashyn et al. 2024). Completeness was evaluated using Benchmarking Universal Single-Copy Orthologs (BUSCO v5; Manni et al. 2021) with fabales_odb as reference for legumes and embryophyta_odb for outgroup species (Table S2). Only proteomes with <5% missing core orthologs were considered complete and retained for further analysis.

### NLR Identification and Classification

NLRs were extracted from each proteome using NLRtracker v1.3.1 (Kourelis et al. 2021). For accurate class assignment, NLRtracker NB-ARC output files were aligned with RefPlantNLR extracted NB-ARC sequences from supplemental datasets 9, 13, and TN_OTHER (Kourelis et al. 2021).

Sequence alignments were performed using Clustal Omega v1.2.2 (Sievers and Higgins 2014) with default parameters in Geneious Prime v2025.2.1 (https://www.geneious.com). Approximately maximum likelihood phylogenetic trees were generated using FastTree v2.1.11 (Price et al. 2010) with default settings and pseudo-count settings for highly gapped sequences. Trees were rooted using non-plant NLRs from the RefPlantNLR dataset (Kourelis et al. 2021).

Phylogenetic trees were visualized using iTOL (Letunic and Bork 2024). NLR classes were assigned based on monophyletic clustering with respective reference NLRs from RefPlantNLR (Kourelis et al. 2021). For example, CC_R_-NLRs were identified by clustering with NRG1 and ADR1 reference sequences. Complete classification details are provided in Table S3.

Alternative splicing variants were identified through genome localization analysis and removed to count only one NLR per locus (Table S4). NLR abundance was normalized by dividing the total NLR count by the number of protein-coding loci in each genome (Sekhwal et al. 2015) (Tables S4 and S5). Correlation between proteome size and NLR number was assessed using Pearson's correlation coefficient. Statistical analyses were performed in R using custom scripts available at https://github.com/MaravilhaRM/NLR_Classes_MaravilhaR.git and Marques et al. (2026).

### Plant Material and Growth Conditions

For tissue expression analysis, we selected 7 closely related species from Hologalegina and Phaseoloid clades with their corresponding genome assemblies and annotations: *Lathyrus sativus* LS007 (JIC_Lsat_v2.1.1; GCA_963859935.3), *Pisum sativum* Caméor (CAAS_Psat_ZW6_1.0; GCA_024323335.2), *Lens culinaris* CDC Redberry (Lcu.2RBY), *Cicer arietinum* kabuli variety (genotype CDC Frontier; GCA_000331145.1), *Medicago truncatula* A17 (GCF_003473485.1_MtrunA17r5.0-ANR-EGN-r1.9; NCBI Medicago truncatula Annotation Release GCF_003473485.1-RS_2025_04), *Glycine max* Williams 82 (Wm82.a4.v1; GCA_000004515.5), and *Phaseolus vulgaris* genotype G19833 (phavu.G19833.gnm2.ann1.PB8d; GCA_000499845.1). Plants were cultivated in 0.5 L pots containing a mixture of 50% soil, 25% vermiculite, and 25% peat in controlled environment chambers at 22 °C with a 12/12-h photoperiod.

### RNA Extraction and Sequencing

Leaf and root tissues were harvested separately from plants with 3 fully expanded leaves, immediately frozen in liquid nitrogen, and stored at −80 °C until processing. Plants showed spontaneous nodulation upon harvest. Total RNA was extracted from approximately 100 mg of tissue using the GeneJET Plant RNA Purification Mini Kit (Thermo Scientific, Vilnius, Lithuania) according to the manufacturer's protocols, with 3 biological replicates per tissue and species.

RNA samples were treated with Turbo DNase I (Ambion, Austin, TX, USA) following manufacturer's instructions. RNA concentration was quantified using a Qubit 2.0 Fluorometer (Invitrogen, Life Technologies, CA, USA) with Qubit RNA BR Assay Kit. RNA purity was assessed by measuring absorbance ratios at 260/280 nm and 260/230 nm using a NanoDrop 2000c spectrophotometer (Thermo Scientific, Passau, Germany). RNA integrity was verified by electrophoresis on 1% agarose gels stained with SYBR Safe (Life Technologies, CA, USA) at 100 V for 30 min.

RNA samples were submitted for Illumina NovaSeq 6000 Sequencing PE150 (40 million paired end reads per sample) at Novogene (UK) Co. Raw sequence data quality was assessed using FastQC (Andrews 2010). Read preprocessing included removal of the first 10 base pairs corresponding to barcode contamination, removal of Illumina Universal adapters, and trimming of bases with Phred quality scores <20 using cutadapt v4.0 (Martin 2011).

### RNA-seq Data Analysis

Processed reads were aligned to the corresponding reference genomes used for NLR discovery using HISAT2 v2.2.1 (Kim et al. 2015). SAMtools v1.9 (Li et al. 2009) was used for BAM file sorting and conversion. Transcript quantification was performed using StringTie v2.2.1 (Pertea et al. 2016). Genes with average expression <5 transcripts across all samples and alternative splicing variants were excluded from analysis.

Count data normalization was performed using the trimmed mean of M-values (TMM) method (Table S6). Tissue specificity assessment involved averaging expression values across leaf and root tissue replicates. Genes were classified as tissue-specific if they showed significant expression (average TMM >0.5 across 3 replicates) in one tissue while remaining lowly expressed (average TMM <0.5) in the other tissue (Table S7).

Differential expression analysis was conducted in R using DESeq2 with root samples as the reference (Anders and Huber 2010; Love et al. 2014). DEGs between leaf and root tissues were identified using a false discovery rate threshold of 0.05 and a log_2_ fold change |>1| (Table S8).

To test whether NLRs are over-represented among genes differentially expressed between leaf and root relative to the genome-wide background, for each species, we cross-tabulated expressed genes by NLR status and by differential expression (using the thresholds above) in a 2 × 2 contingency table and applied a 2-tailed Fisher's exact test for each species, adjusting the *P*-values across species with the Benjamini–Hochberg procedure and estimating a common odds ratio with a Cochran–Mantel–Haenszel test stratified by species (Table S8).

### Phylogenetic Analysis of NLR Subclasses

Phylogenetic analyses of expressed NLRs were performed using a combination of Geneious Prime v2025.2.1 with Clustal Omega alignment tool v1.2.2 (Sievers and Higgins 2014) and FastTree plugin v2.1.11 (Price et al. 2010). NB-ARC domains from the expressed NLRs were aligned with the RefPlantNLR extracted NB-ARC domain dataset and phylogenetic trees constructed as described above to investigate clustering patterns with reference NLRs. NLR subclasses were extracted based on branch distances (0.315 for TNPs, 1.22 for CC_G10_-NLRs, 1.25 for CC_R_-NLRs, 1.2 for CC-NLRs, and 1.24 for TIR-NLRs) per class subtree (Table S9). NLR subclass characteristics (number of NLRs, number of species present, and different expression classes) were summarized in Table S10.

## Supplementary Material

evag257_Supplementary_Data

## Data Availability

The raw RNA-seq data and corresponding metadata generated in this study have been deposited in the ArrayExpress database at EMBL-EBI under accession number E-MTAB-15524. All NLR annotation files, data, and related analysis outputs are available at Zenodo https://doi.org/10.5281/zenodo.18173999 (Marques et al. 2026). All other data supporting the findings of this study are available within the manuscript and its supplementary information files. All custom scripts used for analysis are available at https://github.com/MaravilhaRM/NLR_Classes_MaravilhaR.
